# A comparative study of gradient nonlinearity correction strategies for processing diffusion data obtained with ultra‐strong gradient MRI scanners

**DOI:** 10.1002/mrm.28464

**Published:** 2020-10-03

**Authors:** Umesh Rudrapatna, Greg D. Parker, Jamie Roberts, Derek K. Jones

**Affiliations:** ^1^ Cardiff University Brain Research Imaging Centre Cardiff University Cardiff United Kingdom; ^2^ Royal United Hospitals Bath NHS Foundation Trust Bath United Kingdom; ^3^ Mary MacKillop Institute for Health Research, Faculty of Health Sciences Australian Catholic University Melbourne Victoria Australia

**Keywords:** connectom, diffusion MRI, diffusion preprocessing, gradient nonlinearity, human connectome project, ultra‐strong gradients

## Abstract

**Purpose:**

The analysis of diffusion data obtained under large gradient nonlinearities necessitates corrections during data reconstruction and analysis. While two such preprocessing pipelines have been proposed, no comparative studies assessing their performance exist. Furthermore, both pipelines neglect the impact of subject motion during acquisition, which, in the presence of gradient nonlinearities, induces spatio‐*temporal* B‐matrix variations. Here, spatio‐temporal B‐matrix tracking (STB) is proposed and its performance compared to established pipelines.

**Methods:**

Diffusion tensor MRI (DT‐MRI) was performed using a 300 mT/m gradient system. Data were acquired with volunteers positioned in regions with pronounced gradient nonlinearities, and used to compare the performance of six different processing pipelines, including STB.

**Results:**

Up to 30% errors were observed in DT‐MRI parameter estimates when neglecting gradient nonlinearities. Moreover, the order in which $B_{0}$ inhomogeneity, eddy current and gradient nonlinearity corrections were performed was found to impact the consistency of parameter estimates significantly. Although, no pipeline emerged as a clear winner, the STB approach seemed to yield the most consistent parameter estimates under large gradient nonlinearities.

**Conclusions:**

Under large gradient nonlinearities, the choice of preprocessing pipeline significantly impacts the estimated diffusion parameters. Motion‐induced spatio‐*temporal* B‐matrix variations can lead to systematic bias in the parameter estimates, that can be ameliorated using the proposed STB framework.

## Abstract 2

Click here for author‐reader discussions

## 1 INTRODUCTION

**FIGURE 1:**
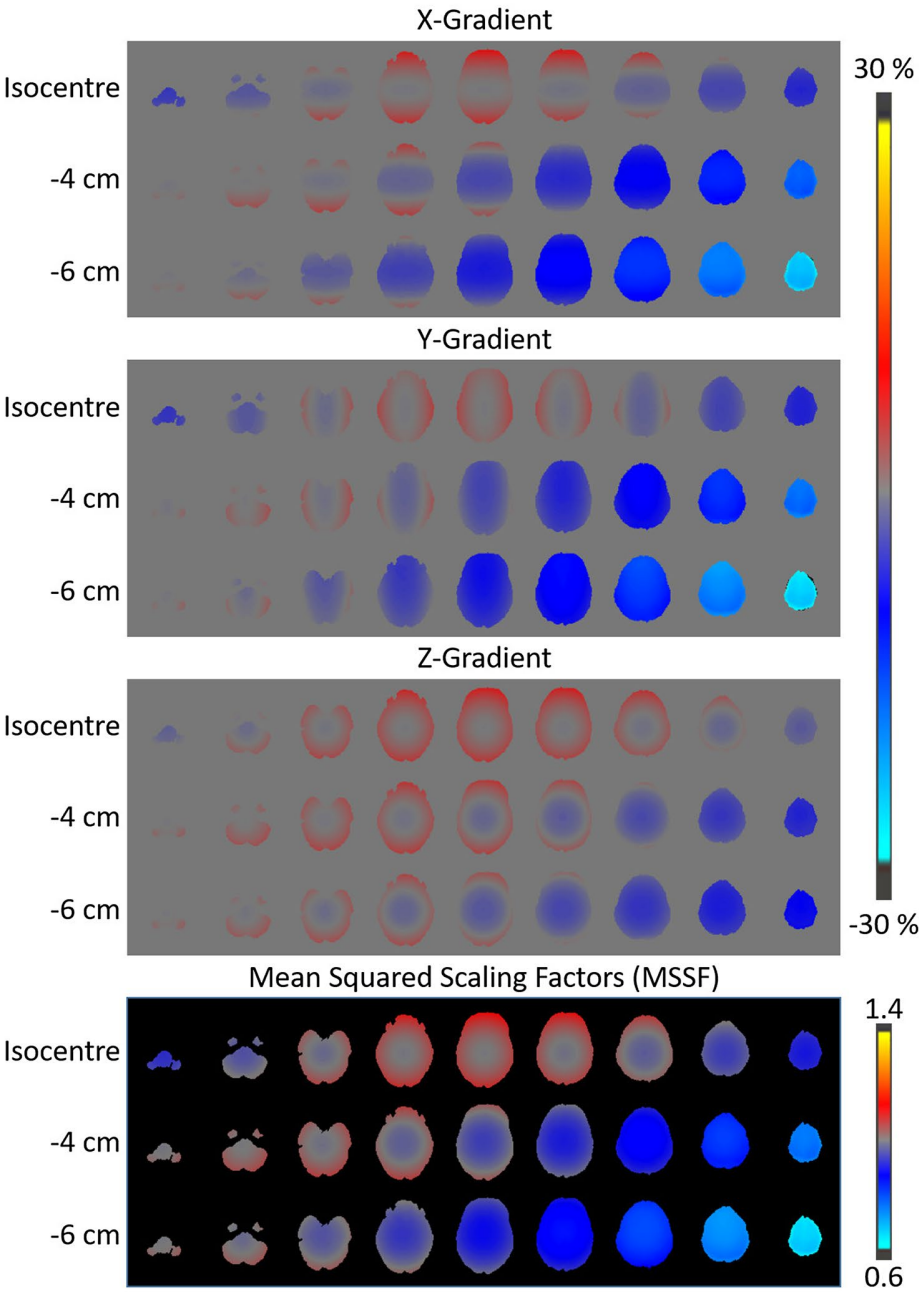
Volunteers were scanned using a diffusion MRI protocol on a 300 mT/m Connectom scanner with different bed translations. *X*, *Y* and *Z* gradient nonlinearities from one of the subjects is presented here for a subset of bed translations. As the volunteers were moved further into the scanner, the gradient nonlinearity effects became more severe and reached nearly −30% of nominal value. The mean of the square of gradient scaling factors (MSSF) due to gradient nonlinearity from the same volunteer is provided at the bottom of the figure and shows significant MSSF variations across bed positions

Recent developments in gradient technology have opened up immense opportunities to further our understanding of brain microstructure.^1^, ^2^, ^3^ However, ultra‐strong gradient systems often have compromised gradient linearity.^1^, ^2^ Figure 1 illustrates the typical gradient nonlinearities observed with the 300 mT/m gradient Connectom scanner used in this study. Even with careful head positioning, parts of the brain furthest from the isocenter can experience significant differences in gradient amplitude (>5%), leading to image distortion and deviations from the prescribed b‐values (which scale with gradient amplitude squared). To utilize the full benefits offered by these gradients, beyond careful head‐positioning, attention has to be given to this often‐neglected confound in the diffusion data processing pipeline.

Aside from gradient nonlinearity, $B_{0}$ inhomogeneity and eddy‐currents also distort the diffusion images, and subject motion compounds their interaction even further. Currently there is no preprocessing pipeline to disentangle their *combined* influence on image distortion.^4^ Though there are effective tools to address each of these distortion sources separately,^5^, ^6^, ^7^, ^8^ the optimal order in which to apply them during preprocessing is unclear. Two preprocessing pipelines have been proposed by the Human Connectome Project (HCP) consortium,^4^, ^9^ with the first advising $B_{0}$ and eddy‐current and motion correction first, followed by gradient nonlinearity distortion correction. The second suggests the opposite order, beginning with gradient nonlinearity distortion correction. Our first objective was to determine which of these pipelines is better suited for processing diffusion MRI data from the 300 mT/m Siemens Connectom scanner.^1^, ^3^ We denote these two pipelines with prefixes WU and MGH, to recognize that the recommendations come from the WASHU‐UMINN HCP project and the MGH‐USC HCP project, respectively. Even if an ideal preprocessing pipeline that addresses all the confounds listed above were to be developed, as noted above, gradient nonlinearities also lead to spatial variations in diffusion weighting. While this is benign for head scanning with most clinical scanners, where spatial uniformity of gradient amplitude is more achievable, the strong gradient nonlinearities in bespoke ultra‐strong gradient systems has prompted the development of dedicated diffusion data analysis pipelines^10^ that account for gradient‐nonlinearity‐induced spatial variations in B‐matrices.^8^, ^11^, ^12^, ^13^, ^14^, ^15^ Here, we consider for the first time, the *interaction* of subject motion with gradient nonuniformity on diffusion measurements, as the effect of diffusion‐weighting cannot be captured using spatially varying B‐matrices alone. Under such circumstances, *spatio‐temporal tracking of B‐matrix* at each voxel location is essential.

The second objective of this work was, therefore, to develop a pipeline that also corrects for these temporal B‐matrix changes, and to compare its performance to the current state‐of‐the‐art.

## 2 METHODS

Diffusion MRI datasets were collected from six healthy volunteers (23‐39 years, 2 female) on a 3T, 300 mT/m Siemens Connectom scanner. The study was approved by the Cardiff University School of Psychology Ethics Committee. A 32‐channel head coil was used for all the experiments. The scan settings were‐ Field of view: Transversal 220 × 220 × 140 ${\mathrm{mm}}^{3}$, 2 × 2 × 2 ${\mathrm{mm}}^{3}$ resolution, TR: 2700 ms, TE: 45 ms, GRAPPA and multi‐band factor 2, b = 1200 s/${\mathrm{mm}}^{2}$, 61 spherically distributed B‐matrix.^16^ Four volunteers (Subjects 2‐5) were scanned five times, with the mid‐sagittal corpus callosum positioned at the isocenter (best estimate) and then, at −3, −4, −5 and −6 cm from isocenter (into the scanner bore). One volunteer (Subject 1) was scanned at −8 cm offset instead of −5 cm. For Subject 6, scanning was skipped at −5 cm offset. At each position, an additional b = 0 s/${\mathrm{mm}}^{2}$ scan was performed with reversed phase encoding for $B_{0}$‐inhomogeneity‐induced distortion correction.^6^ $B_{0}$ shimming was performed at each bed translation separately before starting the diffusion scan. The volunteer’s heads were padded in the receiver coil to restrain movement. However, subjects 5 and 6 were instructed to deliberately move their head continuously during the off‐isocenter scans. The gradient nonlinearities at each bed translation were calculated using the vendor‐provided spherical harmonic coefficients.

Figure 1 shows the nonlinearities of the three gradient coils and the Mean of the squared values of gradient Scaling Factors (MSSF) observed in brain regions at a subset of bed translations in one of the subjects. MSSF is defined as $({\left(1+\delta G_{x}\right)}^{2}+{\left(1+\delta G_{y}\right)}^{2}+{\left(1+\delta G_{z}\right)}^{2})/3$, where $\delta G_{x}$, $\delta G_{y}$ and $\delta G_{z}$ are signed relative gradient deviations from ideal, expressed as a fraction of unity, and are obtained from the spherical harmonic coefficients that represent the gradient nonlinearities. An offset as little as 3 cm from the isocenter can result in more than 5% deviation in *X* or *Y* gradient amplitudes, highlighting the importance of careful head positioning. With bed translations of 5 and 6 cm, the superior brain regions experience more than 30% deviation from their prescribed values.

DT‐MRI parameter estimates were obtained with six different preprocessing pipelines, all of which included $B_{0}$ inhomogeneity correction,^6^ motion and eddy current correction,^7^ gradient nonlinearity distortion correction (GDC)^5^ and either B‐matrix rotation (BR),^17^ which is also included in the FSL eddy tool^7^ with or without spatial B‐matrix correction (SB),^8^ or spatio‐temporal B‐matrix tracking (STB) proposed here.^18^

The six preprocessing pipelines are summarized below:

1. MGH‐BR: GDC ⇒ FSL topup, eddy ⇒ DT‐MRI fit with BR.
2. CUBRIC‐STB1 (Proposed): GDC ⇒ FSL topup, eddy ⇒ DT‐MRI fit with STB.
3. WU‐BR: FSL topup, eddy ⇒ GDC ⇒ DT‐MRI fit with BR.
4. CUBRIC‐STB2 (Proposed): FSL topup, eddy ⇒ GDC ⇒ DT‐MRI fit with STB.
5. MGH‐SB^9^: MGH‐BR and SB.
6. WU‐SB^4^: WU‐BR and SB.

The most commonly used processing pipeline for diffusion datasets is MGH‐BR. When correcting for motion that includes head rotation, it is important that any rotations of the image are also applied to the encoding matrix (B‐matrix), to preserve the relationship between encoding direction and signal intensity. The so‐called “B‐matrix” rotation^17^ is the most commonly used technique. However, this approach implicitly assumes that the gradients are all linear over the field of view of interest. The spatial B‐matrix correction (SB)^8^ rectifies this assumption by accounting for spatial variations in b‐values due to gradient nonlinearities. When a subject moves, the gradient amplitudes experienced by different parts of the brain also change over time, leading to different diffusion weightings. We hypothesize that our proposed spatio‐*temporal* B‐matrix tracking (STB) captures and uses such B‐matrix variations to quantify diffusion metrics more accurately, and we attempt to verify this hypothesis in this work.

The proposed pipelines (CUBRIC‐STB1 and CUBRIC‐STB2) use the motion parameters estimated by FSL *eddy* for STB. Given that gradient nonlinearity is stationary in time, the motion parameters provided by the *eddy* tool can be used to map the temporal evolution of gradient amplitudes for every voxel location. From this, a specific B‐matrix is calculated for each voxel, at each diffusion‐weighting volume and used to estimate voxel‐wise diffusion metrics.

For each pipeline, the FA (fractional anisotropy), MD (mean diffusivity), AD (axial diffusivity) and RD (radial diffusivity) parameters obtained from DT‐MRI fitting at different bed translations were co‐registered to the reference isocenter maps using ANTS(Syn).^19^ The underlying gradient nonlinearities were also transformed to the reference space. The resulting co‐registered data were used to assess percentage change in DT‐MRI parameters with respect to the parameters obtained from data acquired at the isocenter. Finally, to determine which preprocessing pipeline was most robust, that is, least sensitive to gradient nonlinearities induced through bed translations and/or subject motion, statistical analyses were performed on the coefficient of variation (COV) of DTI indices computed voxel‐wise across different bed translations.

## 3 RESULTS

**FIGURE 2:**
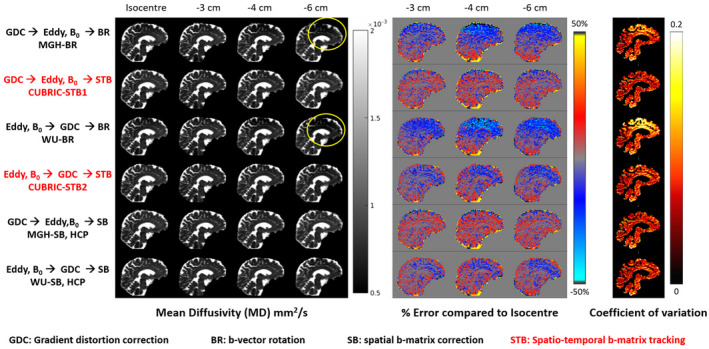
Grayscale image: Representative MD values in a volunteer estimated using different processing pipelines with data acquired at different bed translations. This volunteer (Subject 6) moved significantly during data acquisition. Please refer to Supporting Information Figs. S4, S5 for motion‐related information of all subjects. Pipelines MGH‐BR and WU‐BR, which do not take into account gradient nonlinearities in parameter estimation, severely under‐estimated MD in the frontal and superior‐cortical regions (marked with yellow ellipses), where gradient nonlinearity effects were most severe. Color image block in the middle: % Error in MD estimates relative to isocenter values. Even at 3 cm offset from isocenter, MGH‐BR and WU‐BR underestimate MD by up to 30% and progressively worsen as gradient nonlinearity increases. CUBRIC‐STB1 and MGH‐SB maintain near‐identical performance despite severe gradient nonlinearities. This is also endorsed by the last color image block which shows COV calculated over different bed translations for each pipeline, after ignoring CSF voxels (MD $>0.9\;\times \;10^{‐3}\;{\mathrm{mm}}^{2}$/s)

Figure 2 shows MD values estimated from data obtained at various bed translations using different pipelines and the corresponding COV maps. Pipelines that ignore the impact of gradient nonlinearities on the B‐matrix (MGH‐BR and WU‐BR) fail to replicate MD estimates, while pipelines that account for spatial B‐matrix changes are relatively immune to such discrepancies.

To assess the impact of different preprocessing and DT‐MRI fitting strategies, we calculated the MD values at different bed translations. Only those voxels with gradient deviation >10% in at least one of the gradient axes at any bed position were considered for this analysis, and we excluded voxels representing CSF by thresholding the MD values that exceeded $0.9\;\times \;10^{‐3}\;{\mathrm{mm}}^{2}$/s. This left around 20% of all brain voxels for further analysis in every subject. Even at just 3 cm offset from isocenter, MGH‐BR and WU‐BR underestimate MD by up to 30% in some parts of the brain.

**FIGURE 3:**
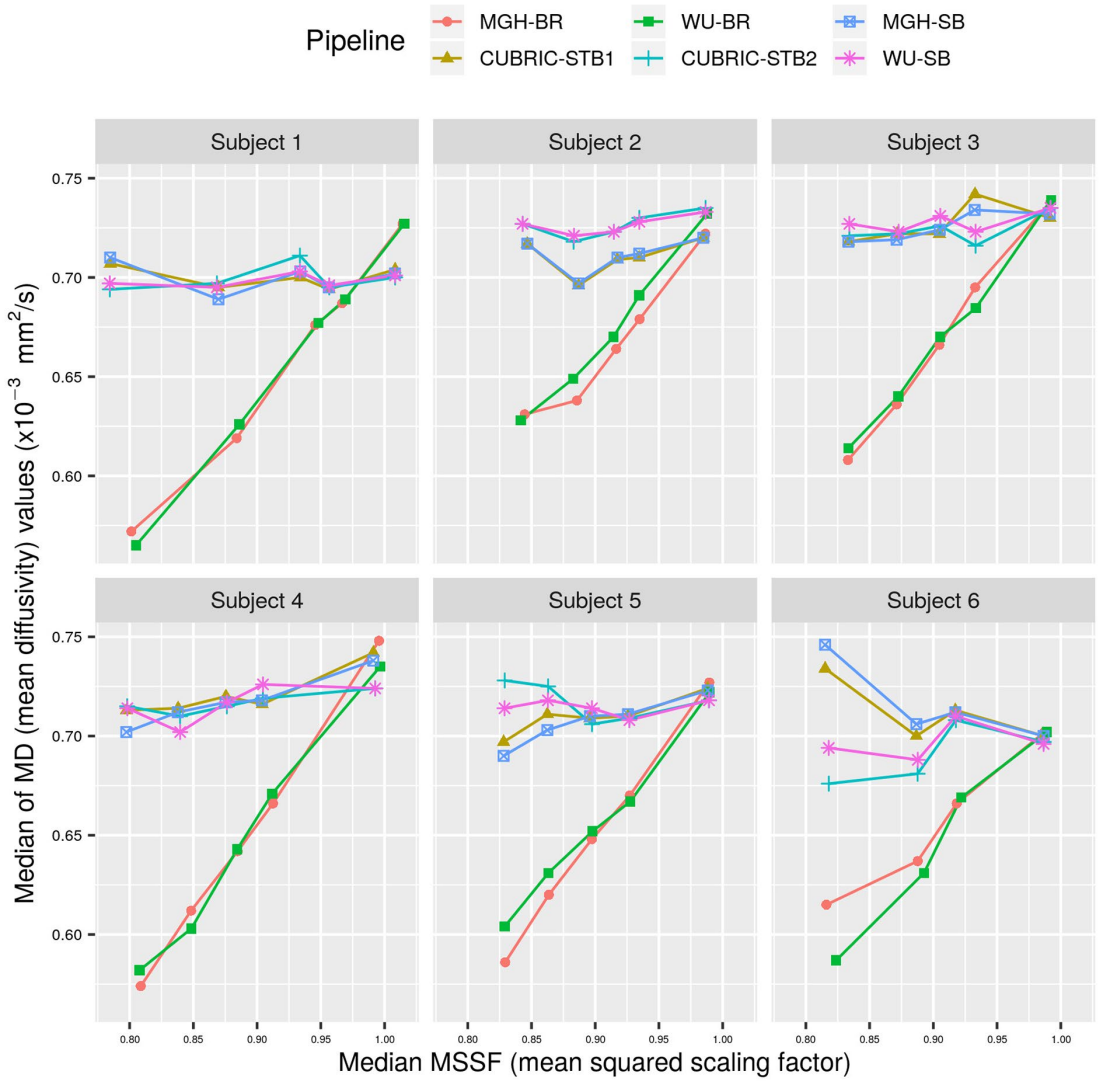
Voxels where at least one of the gradients deviated by more than 10% during bed translations were selected and the median MD value of all such voxels is plotted here against the median MSSF values. Around 20% of brain volume excluding CSF were available in each subject for this analysis. MGH‐BR and WU‐BR which do not take into account gradient nonlinearities in parameter estimation show a near linear trend with MSSF, while other techniques are fairly robust against it. Linear regression tests showed that MGH‐BR and WU‐BR showed significant (*P*<.05) linear trends with MSSF. Results from the analysis of Axial Diffusivity (AD), Radial Diffusivity (RD), and Fractional Anisotropy (FA) from the same voxels are provided in Supporting Information Figures S1‐S3, respectively

We estimated the median MD value from all the selected voxels for each subject and each pipeline and plotted them against the median MSSF values calculated from the same voxels (Figure 3). Pipelines that ignore the impact of gradient nonlinearities on the b‐values (MGH‐BR and WU‐BR) showed a nearly linear trend between the MD values and MSSF. This is expected, since b‐value scales with squared gradient amplitude, and thus, an assumed higher/lower b‐value will lead to an underestimation/overestimation of MD values compared to the ground truth. Pipelines which correct for gradient nonlinearities should show consistent estimates of MD values irrespective of the gradient nonlinearities, and thus bed translations. Thus, an ideal pipeline would give a constant MD value over the whole range of median MSSF values in Figure 3.

**FIGURE 4:**
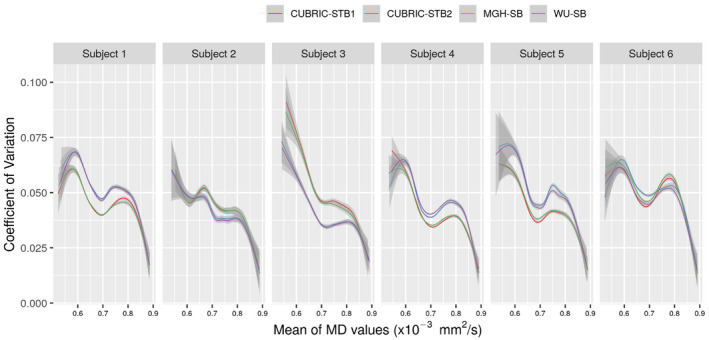
Coefficient of Variation (COV) in MD values for four different pipelines (across bed translations) for all the six subjects. Only four pipelines have been compared here, as the performance of MGH‐BR and WU‐BR were clearly inferior compared to these pipelines, as observed from Figures 2, 3 and Table 1. The plots represent the smoothed conditional means obtained using R package ggplot2,^20^ with the gray bands indicating the 95% confidence interval in the estimated mean values

For the same voxels, we calculated the COV in MD values obtained from different bed translations. Table 1 lists the mean COV for each of the six pipelines for each subject. Furthermore, to understand the behavior of the different pipelines, we plotted the variation of COV across different MD values for the four pipelines that include a correction for gradient nonlinearities (Figure 4).

**TABLE 1:** Summary of the mean COV of DT‐MRI metrics calculated across different bed translations for all the subjects and pipelines

Subject	Voxels	MGH‐BR	CUBRIC‐STB1	WU‐BR	CUBRIC‐STB2	MGH‐SB	WU‐SB
*MD*							
1	1031170	0.1030	**0.0435**	0.1100	0.0511	**0.0433**	0.0506
2	196770	0.0794	0.0450	0.0740	**0.0410**	0.0452	**0.0402**
3	280085	0.0854	0.0472	0.0780	**0.0373**	0.0463	**0.0369**
4	1059655	0.1040	**0.0369**	0.102	0.0418	**0.0375**	0.0430
5	440245	0.0867	**0.0396**	0.0797	0.0478	**0.0406**	0.0464
6	538168	0.0717	**0.0485**	0.0838	0.0513	0.0503	**0.0477**
All		0.0916	**0.0424**	0.0938	0.0465	**0.0429**	0.0459
*AD*							
1		0.1020	**0.0575**	0.1081	0.0633	**0.0573**	0.0635
2		0.0851	0.0533	0.0788	**0.0492**	0.0536	**0.0485**
3		0.0929	0.0572	0.0840	**0.0485**	0.0567	**0.0481**
4		0.1071	**0.0490**	0.1067	0.0555	**0.0487**	0.0562
5		0.0933	**0.0502**	0.0878	0.0575	**0.0508**	0.0562
6		0.0863	**0.0649**	0.0962	0.0670	0.0665	**0.0642**
*RD*							
1		0.1008	**0.0556**	0.1091	0.0628	**0.0550**	0.0624
2		0.0787	0.0507	0.0735	**0.0464**	0.0508	**0.0460**
3		0.0833	0.0538	0.0754	**0.0430**	0.0526	**0.0430**
4		0.1034	**0.0449**	0.1026	0.0517	**0.0452**	0.0526
5		0.0840	**0.0457**	0.0771	0.0571	**0.0461**	0.0551
6		0.0713	0.0633	0.0826	**0.0623**	0.0651	**0.0612**
*FA*							
1		**0.1220**	0.1389	**0.1264**	0.1389	0.1379	0.1402
2		**0.1131**	0.1277	**0.1043**	0.1210	0.1286	0.1213
3		**0.1159**	0.1302	**0.1091**	0.1252	0.1310	0.1254
4		**0.1153**	0.1313	**0.1281**	0.1412	0.1289	0.1399
5		**0.1131**	0.1276	**0.1210**	0.1377	0.1258	0.1351
6		**0.1572**	0.1695	**0.1518**	0.1618	0.1702	0.1632

Notes

The MD table was obtained from the same data used for generating Figure 4. The column “Voxels” in the MD table indicates the actual number of voxels in each subject over which the COV statistics were obtained. The same is valid for other tables as well. For each subject, we have highlighted the two pipelines that yielded the lowest mean COV. The last row in MD table listed as “All,” used the combined data from all the subjects. In red, we have highlighted the pipeline with the overall minimum COV for MD.

To assess the impact of motion on various pipelines, we collected the average voxel‐wise root‐mean‐square motion for each diffusion volume as output by the FSL *eddy* tool^7^ (Supporting Information Figures S4 and S5).

## 4 DISCUSSION

### 4.1 On the performance of MGH‐BR and WU‐BR

Despite HCP recommending the MGH‐SB and WU‐SB processing pipelines, in our observation, some users who download the HCP datasets use commonly available diffusion preprocessing tools which do not account for gradient nonlinearities. Our results (Figures 2 and 3) clearly demonstrate that generic preprocessing pipelines like MGH‐BR and WU‐BR can have an adverse impact on the diffusion metrics obtained. This study re‐emphasizes the need to use MGH‐SB or WU‐SB pipelines at least, as confirmed recently by Mesri et al,^15^ who also showed that ignoring gradient nonlinearity in diffusion measurements can alter significance and effect size in group studies, and thus the conclusions drawn from such studies.

### 4.2 Comparisons between MGH‐SB and WU‐SB

We also observe from Figure 4 and Table 1 that among the HCP recommended pipelines (MGH‐SB and WU‐SB), there is no clear overall winner, with MGH‐SB outperforming WU‐SB in Subjects 1, 4, and 5 and WU‐SB outperforming MGH‐SB in subjects 2, 3, and 6. Moreover, we see that the performances are drastically different in each case. While we cannot clearly elucidate the underlying reasons for this behavior, it seems that performance depends on the amount of gradient nonlinearity and/or motion present in the data. Table 1 shows the number of voxels that met the voxel recruitment criterion (>10% gradient nonlinearity in at least one axis at any bed position). This is clearly larger for Subjects 1 and 4 compared to other subjects, possibly indicating that Subjects 1 and 4 were less‐optimally positioned and experienced greater deviations from the prescribed gradient amplitudes. The lower average MD and RD values and higher AD and FA values in Subjects 1 and 4 compared to other subjects (from Figure 3, Supporting Information Figures S1‐S3, respectively), also supports this observation, as it indicates that significant gradient nonlinearity was also present in WM voxels apart from GM voxels in these subjects. In such cases, it makes sense that MGH‐SB which corrects for gradient nonlinearities first outperforms WU‐SB. Since, WU‐SB first corrects for distortions induced by eddy currents and $B_{0}$, it may mistakenly attribute distortions caused by gradient nonlinearities to these other sources. In contrast, WU‐SB may outperform MGH‐SB when the gradient nonlinearities are relatively benign, as perhaps indicated by the fewer voxels listed against Subjects 2 and 3. For Subject 6, because of large motion, distortions due to $B_{0}$ inhomogeneity would have been greater, compared to other subjects, as the $B_{0}$ shims are calculated and fixed based on the initial condition. MGH‐SB could have been adversely impacted by the confounds caused by $B_{0}$ inhomogeneity. However, by virtue of correcting for motion along with $B_{0}$ inhomogeneity first, WU‐SB perhaps outperforms MGH‐SB in Subject 6. These observations naturally lead us to the comparisons between the STB pipelines and their corresponding SB pipelines.

### 4.3 Comparisons between SB and STB pipelines

From Supporting Information Figures S4 and S5, we observe that Subjects 1‐4 showed benign motion during the scans compared to Subject 6. Although both Subject 5 and 6 were instructed to move during the scans, the same figures show that Subject 5 had motion of comparable magnitude to that of Subjects 1‐4. Although it is hard to unequivocally conclude that the amount of motion detected by retrospective image‐based subject motion detection truly represents the actual motion in diffusion scans, taking these image‐derived estimates along with visual inspection of datasets, we feel safe to cluster subjects 1‐5 together as a group with benign motion.

In these subjects with benign motion, we see a clear correlation between the performance of MGH‐SB and CUBRIC‐STB1 and WU‐SB and CUBRIC‐STB2. This is expected, as the STB pipelines are extensions of their corresponding SB pipelines. However, both Figure 4 and Table 1 indicate that while CUBRIC‐STB1 could perform better than MGH‐SB, CUBRIC‐STB2 may marginally underperform compared to WU‐SB. WU‐SB uses the FSL *eddy* tool to try to correct for motion and eddy currents with images distorted by gradient nonlinearity. However, since this tool does not have knowledge about gradient nonlinearity‐induced distortions, the estimates of motion, eddy currents and/or $B_{0}$ inhomogeneity could be compromised. Subsequently, inaccurate motion estimates will impact the accuracy of STB , which may explain the observed underperformance of CUBRIC‐STB2 compared to MGH‐SB. In contrast, MGH‐SB performs motion estimation *after* correction for distortions arising from gradient nonlinearity. Thus, the motion estimates resulting from *eddy* that runs after gradient distortion correction could provide more reliable inputs to STB. This perhaps also explains why CUBRIC‐STB1 seems to perform better than MGH‐SB in the case of Subject 6 with large motion, while CUBRIC‐STB2 seems to underperform compared to WU‐SB under the same conditions.

### 4.4 About the benefits of STB pipelines

One of our objectives was to introduce STB to handle interactions between subject motion and gradient nonlinearities. This brings us to the question of whether there are advantages to choosing STB over SB pipelines. As our results show, with significant gradient nonlinearities at play, CUBRIC‐STB1 seems to show a small improvement over MGH‐SB. Furthermore, although a first glance at Table 1 indicates that in case of severe motion (Subject 6), WU‐SB outperforms CUBRIC‐STB1, a closer look at Figure 4 reveals a more complex picture. For every subject, all the pipelines display a particular COV trend, with a dip in COV values between $0.65\;\times \;10^{‐3}$ and $0.75\;\times \;10^{‐3}\;{\mathrm{mm}}^{2}$/s, which corresponds to roughly the MD range of white matter and gray matter. In Subject 6, 50% of all analyzed voxels had MD values within this range. The MD values above this range could indicate CSF or GM/CSF boundaries (20% of all voxels in Subject 6). As seen in Figure 4, MD values below $0.6\;\times \;10^{‐3}\;{\mathrm{mm}}^{2}$/s have large confidence intervals and could represent very few voxels (10% in case of Subject 6). Thus, we should be able to safely evaluate the performance of the different pipelines within the window of $0.65\;\times \;10^{‐3}$ and $0.75\;\times \;10^{‐3}\;{\mathrm{mm}}^{2}$/s. In this window, from Figure 4, with substantive subject motion (Subject 6), CUBRIC‐STB1 seems to perform better than other pipelines. Moreover, this trend continued when data from all the subjects were pooled together as well (Table 1, MD section). These observations indicate that further investigations on CUBRIC‐STB1 pipeline could yield valuable insights and results. The better performance of CUBRIC‐STB1 on pooled data could perhaps also be due to the greater number of voxels listed against Subjects 1 and 4. Despite these observations, we would like to re‐emphasize that in case of benign gradient nonlinearities and benign motion, WU‐SB could still be the preferred preprocessing pipeline.

### 4.5 Correcting FA values

Table 1 and Supporting Information Figure S3 make it abundantly clear that mitigating the influence of gradient nonlinearities on FA values is much more difficult than addressing MD values. In fact, all additional processing beyond B‐matrix rotation worsened the COV of FA values (Table 1). However, we clearly observe that all the techniques that accounted for gradient nonlinearity in FA calculations showed higher FA values compared to MGH‐BR and WU‐BR (Supporting Information Figure S3), especially in subjects where significant white matter voxels were analyzed (subjects 1, 4, and 6). The difference between the methods was less obvious in subjects where predominantly gray matter voxels were analyzed (subjects 2, 3, and 5). These observations clearly align with those from Mesri et al,^15^ who reported that scalar parameters could be corrected better than angular parameters and that the effect of gradient nonlinearity correction on FA depended on the tissue type. Clearly, further research is needed to address the large COV observed in FA values.

### 4.6 Limitations of the study

As mentioned in the introduction, one of our objectives was to assess the impact of the order in which preprocessing steps are performed on the final DT‐MRI parameters. This also opens the question of whether it is in fact possible to completely disentangle the effects of $B_{0}$ inhomogeneity, eddy currents, gradient nonlinearity‐induced distortions, $B_{1}$ inhomogeneity and motion using tools that address these confounds sequentially, as a composition of different processing steps. From the image formation perspective, all these non‐idealities act simultaneously. With the exception of subject motion, the remaining non‐idealities can be measured and corrected for, during image reconstruction. The same approach may have to be adopted during preprocessing if not addressed during reconstruction. Currently there are no tools to do this and thus we have restricted our preprocessing options to those that combined widely available and popular choices.

Among the system imperfections, we have tried to minimize the effect of $B_{0}$ inhomogeneity on bed translations through image‐based shimming and by acquiring opposite PE‐encoded images for EPI distortion correction (FSL *topup* tool). Similarly, the FSL *eddy* tool would have mitigated most eddy current effects. To the best of our knowledge, $B_{1}$ variations at different bed positions are relatively benign compared to gradient nonlinearity variations. Specifically, the Connectom scanner’s transmit body coil has a larger coverage than the gradients and thus the variation of transmit efficiency over 8 cm translation, as used here, is, from our experience, relatively minor compared to the gradient nonlinearity variations. Nonetheless, it is possible that the effectiveness of each of these corrections could have been different at different bed positions. Hence, we restricted our comparisons to different processing pipelines and not bed‐positions *per se*. Using the same acquired data for all analyses has allowed for a fair comparison.

In this study, we have used vendor‐provided spherical harmonic coefficients as true gradient nonlinearity measurements in all our analysis. Further methodological improvements can perhaps be obtained by direct empirical measurement of gradient nonlinearity as performed by Lee et al,^14^ which avoids approximations that may result from spherical harmonic expansion.

### 4.7 Conclusions

In this work, our aim was to highlight the need for further research into the impact of the choice of data preprocessing pipeline in diffusion MRI in the presence of significant gradient nonlinearities. We have shown that the optimal choice of processing pipeline is not straightforward and may be conditional on the degree of gradient nonlinearity over the region of interest. Furthermore, we have shown that the interaction between motion and gradient nonlinearities can have significant impact on diffusion metrics and we have introduced the concept of STB to address this issue. Although no single pipeline emerged as the best under all circumstances, our preliminary results seem to indicate that under the circumstances of high gradient nonlinearities and/or high motion, the CUBRIC‐STB1 pipeline could yield more consistent parameter estimates than others.

While we have demonstrated the importance of accounting for gradient nonlinearities using neuroimaging examples in this work, the ideas introduced and discussed here are equally applicable to other anatomical regions and other scanners. For example, large field of view abdominal scans typically suffer from significant gradient nonlinearities in the outermost regions, even in clinical scanners. Accurate diffusion measurements in those regions could also benefit from the STB approach proposed here.

## Supporting information


**FIGURE S1** Same as Figure 3, but with axial diffusivity (AD)
**FIGURE S2** Same as Figure 3, but with radial diffusivity (RD)
**FIGURE S3** Same as Figure 3, but with fractional anisotropy (FA)
**FIGURE S4** Time series of RMS motion per voxel as reported by FSL eddy tool over different diffusion volumes. Subject 6 showed significant movement compared to all other subjects. However, varying degrees of motion can be observed in different subjects at different bed translations
**FIGURE S5** Box plots of RMS motion calculated from the time series shown in Supporting Information Figure S4

## References

[1] Setsompop K , Kimmlingen R , Eberlein E , et al. Pushing the limits of in vivo diffusion MRI for the Human Connectome Project. NeuroImage. 2013;80(Supplement C):220‐233. 23707579 10.1016/j.neuroimage.2013.05.078 PMC3725309

[2] Sotiropoulos SN , Jbabdi S , Xu J , et al. Advances in diffusion MRI acquisition and processing in the Human Connectome Project. NeuroImage. 2013;80(Supplement C):125‐143. 23702418 10.1016/j.neuroimage.2013.05.057 PMC3720790

[3] Jones DK , Alexander D , Bowtell R , et al. Microstructural imaging of the human brain with a ‘super‐scanner’: 10 key advantages of ultra‐strong gradients for diffusion MRI. NeuroImage. 2018;182:8‐38. 29793061 10.1016/j.neuroimage.2018.05.047

[4] Glasser MF , Sotiropoulos SN , Wilson JA , et al. The minimal preprocessing pipelines for the Human Connectome Project. NeuroImage. 2013;80(Supplement C):105‐124. 23668970 10.1016/j.neuroimage.2013.04.127 PMC3720813

[5] Jovicich J , Czanner S , Greve D , et al. Reliability in multi‐site structural MRI studies: effects of gradient non‐linearity correction on phantom and human data. NeuroImage. 2006;30:436‐443. 16300968 10.1016/j.neuroimage.2005.09.046

[6] Andersson JL , Skare S , Ashburner J . How to correct susceptibility distortions in spin‐echo echo‐planar images: application to diffusion tensor imaging. NeuroImage. 2003;20:870‐888. 14568458 10.1016/S1053-8119(03)00336-7

[7] Andersson JL , Sotiropoulos SN . An integrated approach to correction for off‐resonance effects and subject movement in diffusion MR imaging. NeuroImage. 2016;125(Supplement C):1063‐1078. 26481672 10.1016/j.neuroimage.2015.10.019 PMC4692656

[8] Bammer R , Markl M , Barnett A , et al. Analysis and generalized correction of the effect of spatial gradient field distortions in diffusion‐weighted imaging. Magn Reson Med. 2003;50:560‐569. 12939764 10.1002/mrm.10545

[9] Fan Q , Witzel T , Nummenmaa A , et al. MGHG‐USC Human Connectome Project datasets with ultra‐high b‐value diffusion MRI. NeuroImage. 2016;124:1108‐1114. 26364861 10.1016/j.neuroimage.2015.08.075 PMC4651764

[10] The WU‐Minn HCP Open Access Initial Data Release: User Guide and Fact Sheets. Appendix IV—Matlab code for voxel‐wise correction of dMRI gradients; 2012. https://www.humanconnectome.org/storage/app/media/documentation/data_release/October2012_Release_Appendix4.pdf. Accessed October 31, 2012.

[11] Mohammadi S , Nagy Z , Müller HE , et al. The effect of local perturbation fields on human DTI: characterisation, measurement and correction. NeuroImage. 2012;60:562‐570. 22197741 10.1016/j.neuroimage.2011.12.009 PMC3314907

[12] Malyarenko DI , Ross BD , Chenevert TL . Analysis and correction of gradient nonlinearity bias in apparent diffusion coefficient measurements. Magn Reson Med. 2014;71:1312‐1323. 23794533 10.1002/mrm.24773 PMC3823647

[13] Borkowski K , Klodowski K , Figiel H , Krzyżak AT . A theoretical validation of the B‐matrix spatial distribution approach to diffusion tensor imaging. Magn Reson Imaging. 2017;36:1‐6. 27742435 10.1016/j.mri.2016.10.002

[14] Lee Y , Kettinger AO , Wilm BJ , et al. A comprehensive approach for correcting voxel‐wise b‐value errors in diffusion MRI. Magn Reson Med. 2020;83:2173‐2184. 31840300 10.1002/mrm.28078 PMC7065087

[15] Mesri HY , David S , Viergever MA , Leemans A . The adverse effect of gradient nonlinearities on diffusion MRI: from voxels to group studies. NeuroImage. 2020;205:116127. 31476431 10.1016/j.neuroimage.2019.116127

[16] Jones DK , Horsfield M , Simmons A . Optimal strategies for measuring diffusion in anisotropic systems by magnetic resonance imaging. Magn Reson Med. 1999;42:515‐525. 10467296

[17] Leemans A , Jones DK . The B‐matrix must be rotated when correcting for subject motion in DTI data. Magn Reson Med. 2009;61:1336‐1349. 19319973 10.1002/mrm.21890

[18] Rudrapatna SU , Parker GD , Roberts J , Jones DK . Can we correct for interactions between subject motion and gradient‐nonlinearity in diffusion MRI? In Proceedings of the Joint Annual Meeting ISMRM‐ESMRMB 2018; 2018; Paris, France. Program no. 1206.

[19] Avants B , Epstein C , Grossman M , Gee J . Symmetric diffeomorphic image registration with cross‐correlation: evaluating automated labeling of elderly and neurodegenerative brain. Med Image Anal. 2008;12:26‐41. 17659998 10.1016/j.media.2007.06.004 PMC2276735

[20] Wickham H . ggplot2: elegant graphics for data analysis. New York, NY: Springer‐Verlag; 2016. ISBN 978‐3‐319‐24277‐4.

