# Supplementary material for: A comparative study of gradient nonlinearity correction strategies for processing diffusion data obtained with ultra‐strong gradient MRI scanners
Source: Magn Reson Med. 2020 Oct 3;85(2):1104–13. doi: 10.1002/mrm.28464 (PMC8103165; doi:10.1002/mrm.28464)
Supplement: Supplementary file 1 — FIGURE S1 Same as Figure 3, but with axial diffusivity (AD) FIGURE S2 Same as Figure 3, but with radial diffusivity (RD) FIGURE S3 Same as Figure 3, but with fractional anisotropy (FA) FIGURE S4 Time series of RMS motion per voxel as reported by FSL eddy tool over different diffusion volumes. Subject 6 showed significant movement compared to all other subjects. However, varying degrees of motion can be observed in different subjects at different bed translations FIGURE S5 Box plots of RMS motion calculated from the time series shown in Supporting Information Figure S4 [file MRM-85-1104-s001.pdf]

Supporting Information

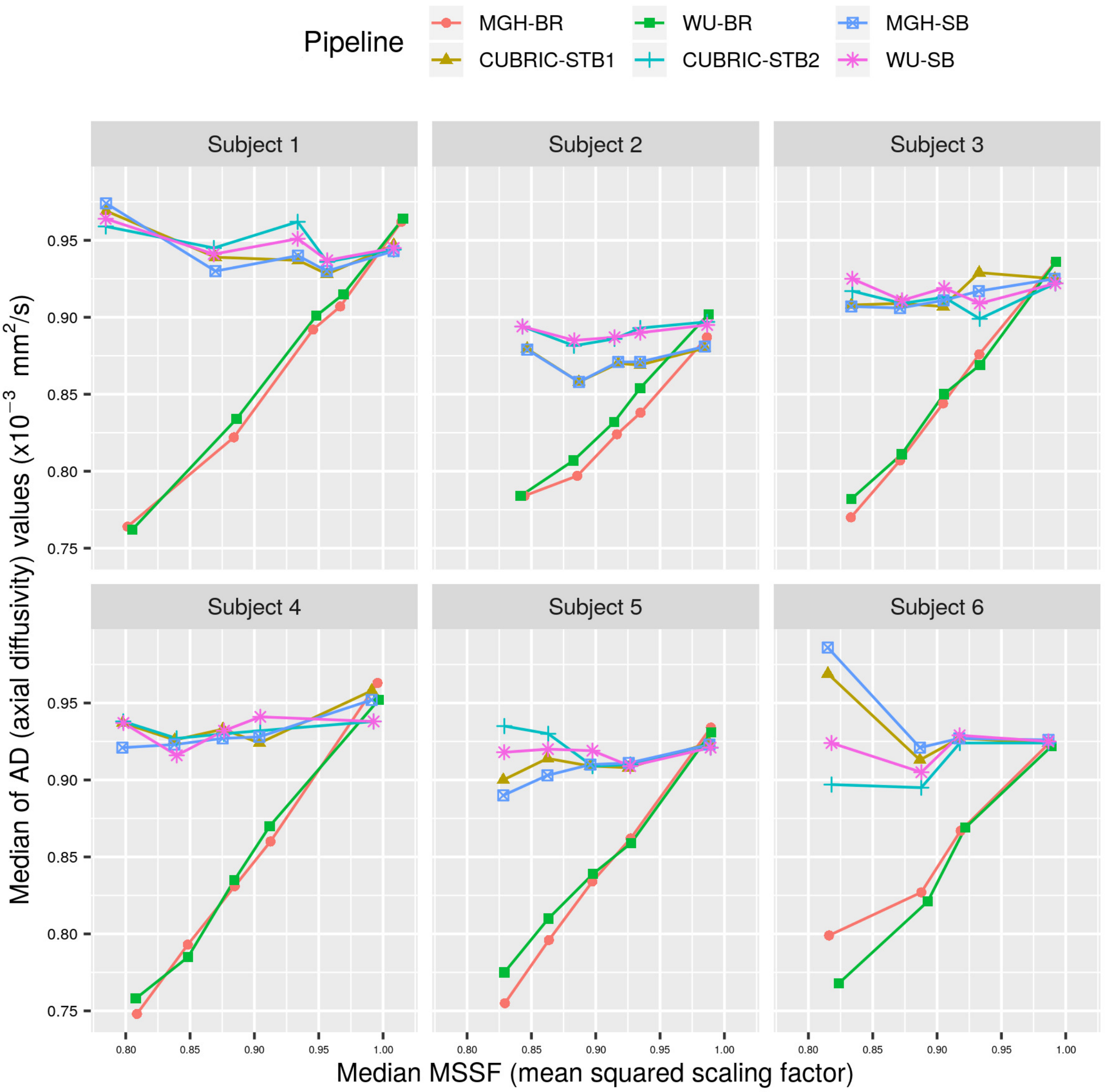

Supporting Information Figure S1: Same as Fig. 3, but with axial diffusivity (AD).

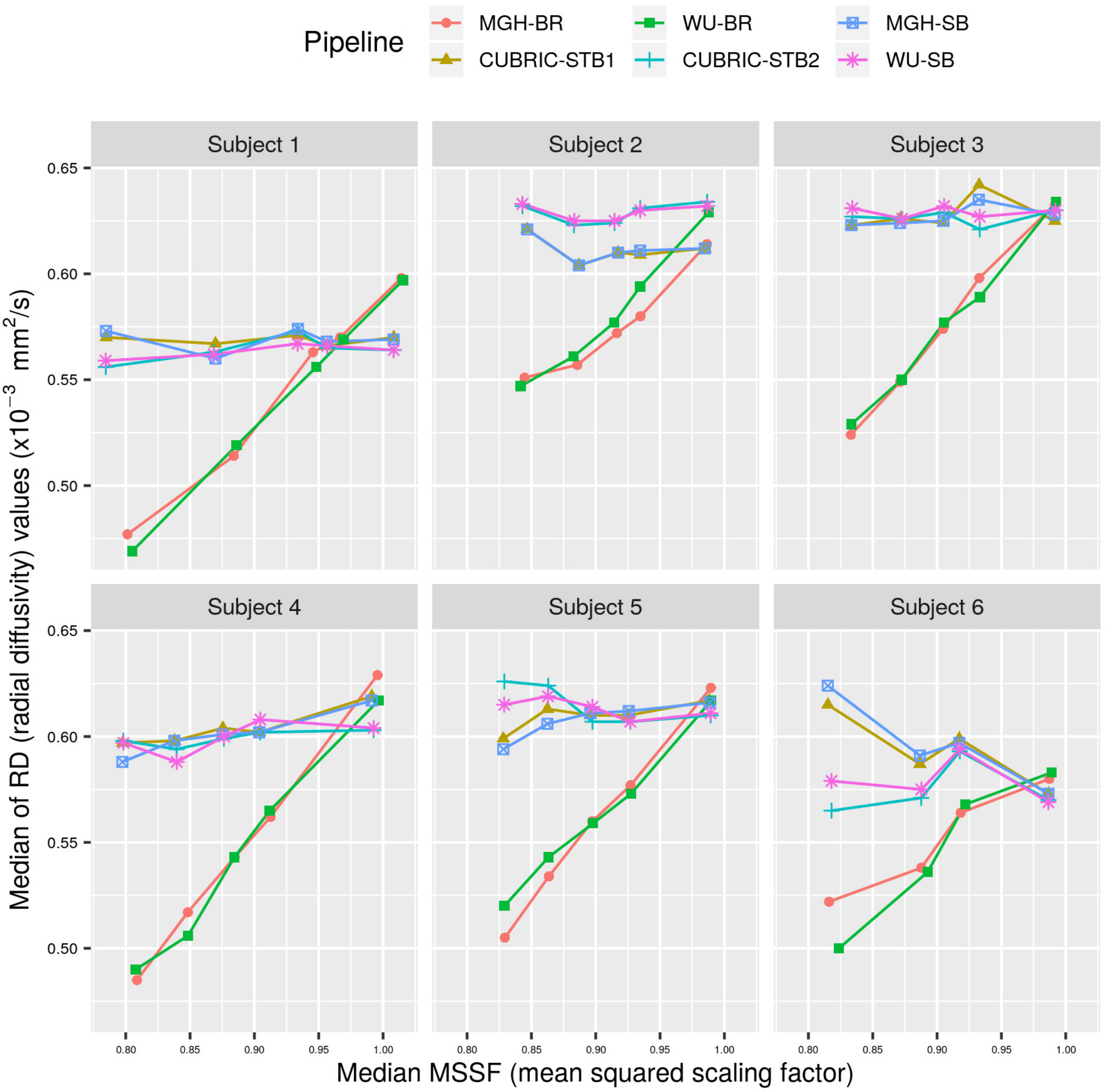

Supporting Information Figure S2: Same as Fig. 3, but with radial diffusivity (RD).

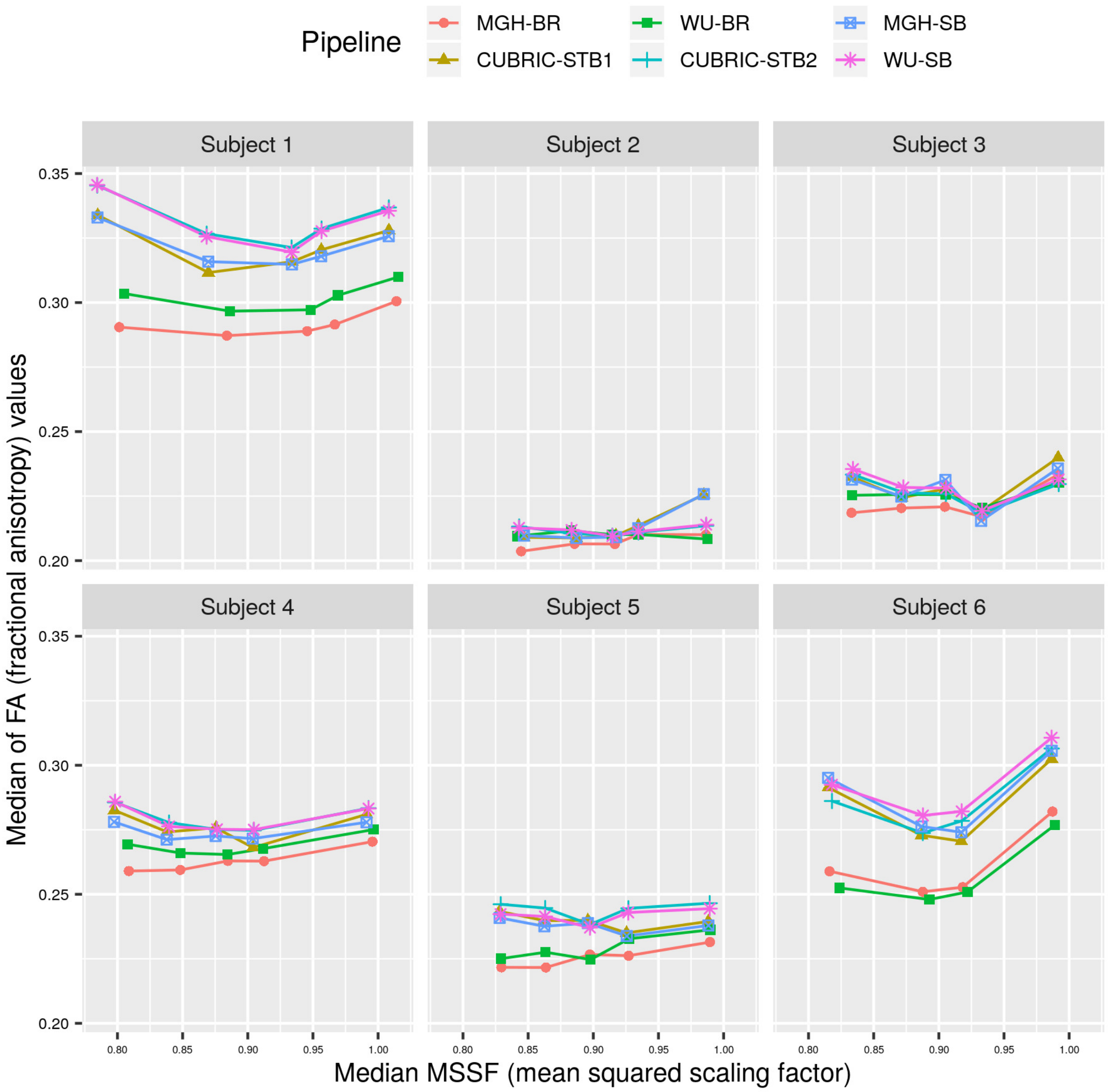

Supporting Information Figure S3: Same as Fig. 3, but with fractional anisotropy (FA).

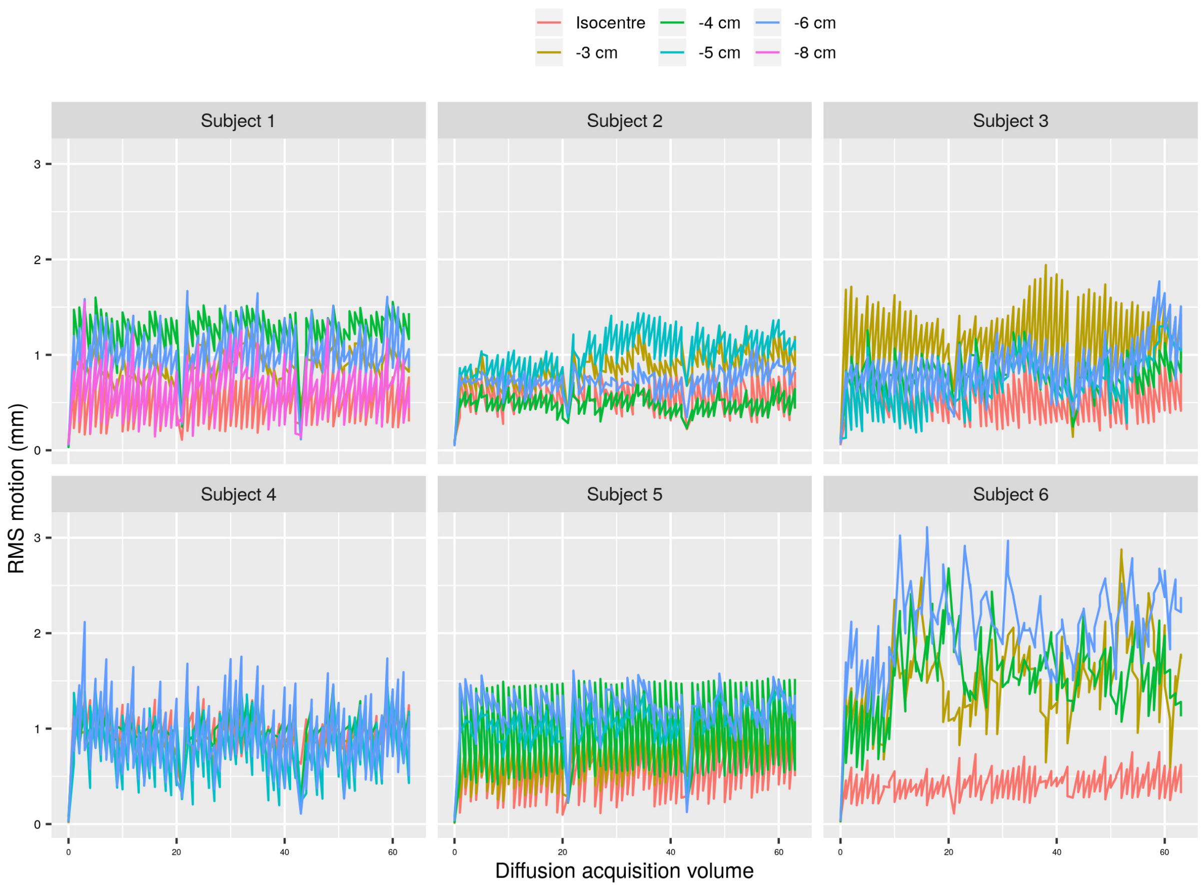

Supporting Information Figure S4: Time series of RMS motion per voxel as reported by FSL eddy tool over different diffusion volumes. Subject 6 showed significant movement compared to all other subjects. However, varying degrees of motion can be observed in different subjects at different bed translations.

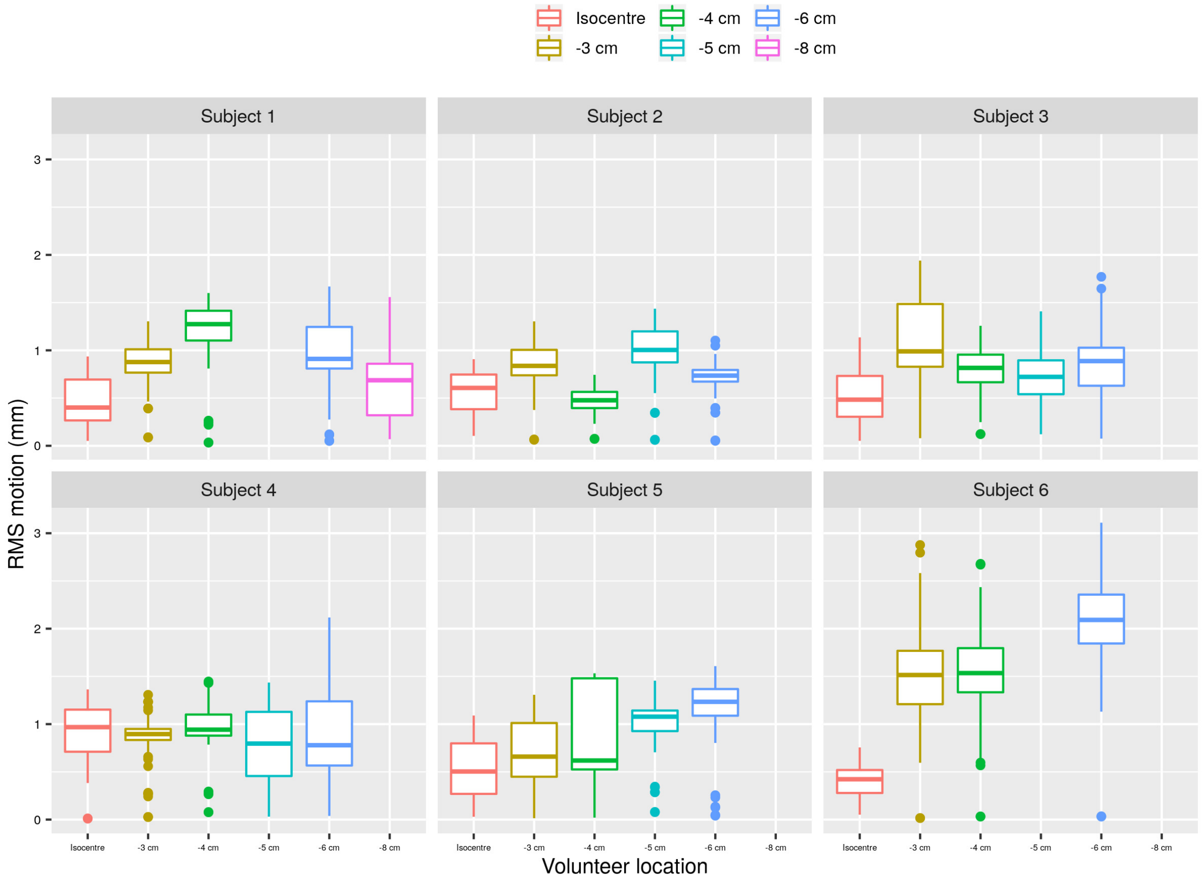

Supporting Information Figure S5: Box plots of RMS motion calculated from the time series shown in Supporting Information Fig. S4.
